# Presynaptic Release-Regulating mGlu1 Receptors in Central Nervous System

**DOI:** 10.3389/fphar.2016.00295

**Published:** 2016-08-31

**Authors:** Anna Pittaluga

**Affiliations:** ^1^Department of Pharmacy, Pharmacology and Toxicology Section, School of Medical and Pharmaceutical Sciences, University of GenoaGenoa, Italy; ^2^Center of Excellence for Biomedical Research, University of GenoaGenoa, Italy

**Keywords:** mGlu1 receptors, transmitter release, autoreceptors, heteroreceptors, presynaptic receptors

## Abstract

Group I metabotropic glutamate (mGlu) receptors consists of mGlu1 and mGlu5 receptor subtypes. These receptors are widely distributed in the central nervous system (CNS), where they preferentially mediate facilitatory signaling in neurones and glial cells, mainly by favoring phospholipase (PLC) translocation. Based on the literature so far available, group I Metabotropic glutamate receptors (mGluRs) are preferentially expressed at the postsynaptic side of chemical synapsis, where they participate in the progression of the chemical stimulus. Studies, however, have shown the presence of these receptors also at the presynaptic level, where they exert several functions, including the modulation of transmitter exocytosis. Presynaptic Group I mGluRs can be both autoreceptors regulating release of glutamate and heteroreceptors regulating the release of various transmitters, including GABA, dopamine, noradrenaline, and acetylcholine. While the existence of presynaptic release-regulating mGlu5 receptors is largely recognized, the possibility that mGlu1 receptors also are present at this level has been a matter of discussion for a long time. A large body of evidence published in the last decade, however, supports this notion. This review aims at revisiting the data from *in vitro* studies concerning the existence and the role of release-regulating mGlu1 receptors presynaptically located in nerve terminals isolated from selected regions of the CNS. The functional interaction linking mGlu5 and mGlu1 receptor subtypes at nerve terminals and their relative contributions as modulators of central transmission will also be discussed. We apologize in advance for omission in our coverage of the existing literature.

## The presynaptic control of transmitter release

Transmitter release is a key event of central transmission. At chemical synapsis, it assures the transmission from the presynaptic to the postsynaptic component of the active synapse, allowing the propagation of the stimulus. The transfer of chemical information from the pre- to the postsynaptic components of the active synapses is mediated by transmitter(s) actively released by the presynaptic side of the synapsis acting at receptors postsynaptically located. Once into the biophase, transmitter(s) also act retrogradely, at receptors located at the synaptic boutons from which they are released, as well as at neighboring terminals. The former ones are called presynaptic receptors and are located within the active zone, while those located close to the synaptic active zone, where vesicular transmitter exit occurs, are referred to as perisynaptic receptors.

**FIGURE 1:**
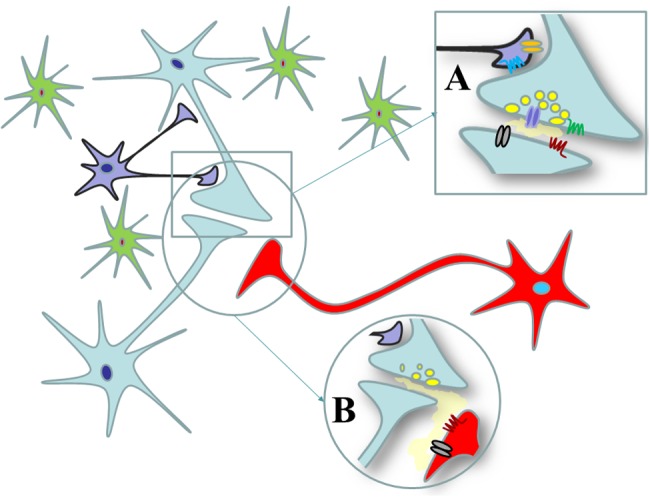
**Synaptic and non-synaptic communications in the central nervous system. (A)** Synaptic communication in central nervous system. Synaptic contacts between nerve endings originating from distinct neurons are organized in chemical synapsis where the presynaptic component releases the transmitter in the synaptic cleft. Once released, the transmitter can act at receptors located at the postsynaptic side of the synapses (black and purple receptors) as well as at autoreceptors located at the presynaptic side (blue and red ones). The transmitter also can target receptors that are located perisynaptically, out of the synaptic active zone (the green and orange receptors). **(B)** Non-synaptic communication in CNS. Nerve terminals also could be connected non-synaptically one to each other. The nerve terminal boutons from the bright green neurons do not make synaptic contact with those originating from the red ones, but they can influence the functions of the latter terminals through the mechanism of the “volume diffusion.” The receptors located on the red terminals are extrasynaptic receptors that are postsynaptically located with respect to the bright green nerve terminals but that are located presynaptically with respect to the red terminals.

Chemical transmission also occurs at neuronal processes that fail to make synaptic contacts. In this case, the non-synaptic compartment senses transmitter release from nerve endings through a mechanism of non-synaptic communication or “volume transmission” (Vizi et al., 2010). The targets of transmitters that diffuse through the “volume transmission” are receptors located at the “non-synaptic release sites,” i.e., those receptors located extrasynaptically (although also receptors existing at the perisynaptic side have been included in this group, Vizi et al., 2010). These “non-synaptic” receptors are located postsynaptically with respect to nerve terminals releasing the transmitter from which they are activated, but they are located presynaptically at nerve edges or varicosities, where they exert their functional effects, including the modulation of transmitter release (**Figure 1**).

At both the synaptic and the non-synaptic sites, depending on the natural ligand that activates the release-regulating receptors, we can distinguish between auto- and heteroreceptors, the former controlling the release of their natural agonist, the latter controlling the release of transmitters other than their natural ligand. Release-regulating receptors can be further classified in negative and positive receptors. Presynaptic negative receptors counter-balance the increased availability of transmitter(s), while presynaptic positive receptors, in particular the autoreceptors, amplify it, then favoring synaptic plasticity, but, if excessive, leading to overt excitotoxicity. Drugs targeting these receptors are suitable candidates as therapeutic agents for restoring altered central neurotransmission.

Of the characterized release-regulating presynaptic receptor systems, the glutamatergic one is particularly complex. Although both ionotropic and metabotropic glutamate (mGlu) receptors modulate transmitter exocytosis, mGlu auto and heteroreceptors predominate as release controllers (Cartmell and Schoepp, 2000; Schoepp, 2001). Metabotropic glutamate receptors (mGluRs) belong to the family 3 of the G protein coupled receptors (GPCRs). By triggering intraterminal enzymatic pathways leading to activation of phosphorylative processes, the group I mGlu receptors positively control the molecular events occurring at nerve terminals. In particular, besides the control of transmitter exocytosis, they can regulate the insertion/internalization of the AMPA and NMDA receptor subunits, determining changes in the subunit composition and, consequently, in receptor-mediated functions. Their structures and activity has been matter of discussion in several reviews (Pin and Bockaert, 1995; Nicoletti et al., 1999, 2011, 2015; Bruno et al., 2001) and will not be further analyzed here.

## The up–down superfusion of a thin layer of synaptosomes: some experimental considerations

In the last decade, great effort has been invested to elucidate whether mGlu1 receptors exist at the presynaptic level and what the functional consequences of their activation are. The majority of the studies were carried out by using synaptosomes in superfusion to monitor transmitter release so that, before focussing on the topic of this review, it seems useful to recall some of the main features of this experimental approach.

Many authors believe that the simplest and well-established model for studying release-regulating receptors is the isolated nerve terminal (synaptosome) preparation. Synaptosomes are freshly isolated nerve terminals that maintain all the functional properties of the originating nerve terminals/varicosities, since they can synthesize, store, release, and re-uptake transmitters. They are endowed with native receptors and transporters, with enzymes and G proteins and contain the cytosolic machinery so that: (i) they synthesize and store transmitters; (ii) they store newly uptaken molecules; (iii) they release transmitters in an exocytotic-like or in a carrier-mediated manner, (iv) they enable physical interaction and functional cross-talk among co-localized receptor proteins, as well as the insertion and deletion of receptor proteins from – to synaptic membranes (v) they favor the transduction of G protein-induced signaling and develop enzymatic processes.

Synaptosomes are isolated from tissue homogenates by centrifugation and then purified on a Percoll gradient to eliminate contaminating particles originating from other cells, including astrocytes. This is particularly relevant in studies dedicated to the pharmacological characterization of presynaptic mGlu1 autoreceptors modulating glutamate release from nerve endings. Actually, although the existence of mGlu1 receptors in astrocytes is matter of discussion (Spampinato et al., 2012 and references therein), the possibility that these receptors are expressed in gliosomes (i.e., particles originating from astrocyte arborisations during tissue homogenisation to isolate synaptosomes, Milanese et al., 2009) deserves attention. If present, glial mGlu1 receptors could elicit glutamate release, then indirectly altering the efficiency of glutamate exocytosis from the synaptosomal suspension. The purification on a Percoll gradient (Dunkley et al., 1986) drastically reduces glial contamination (Milanese et al., 2010), preventing these artifacts.

Raiteri et al. (1974) described for the first time the so-called “up-down superfusion of a thin layer of synaptosomes,” an experimental approach to monitor the release of transmitter from these particles (Raiteri et al., 1974). This technique is nowadays considered a method of choice for the study of presynaptic mechanisms. Two were the main innovations introduced:

(i) Synaptosomes were continuously up-down superfused with a medium thermostated at 37°C having an isoosmotic and isotonic ionic composition with these particles.
(ii) Synaptosomes were layered as a monolayer on porous filters at the bottom of thermostated chambers.

**FIGURE 2:**
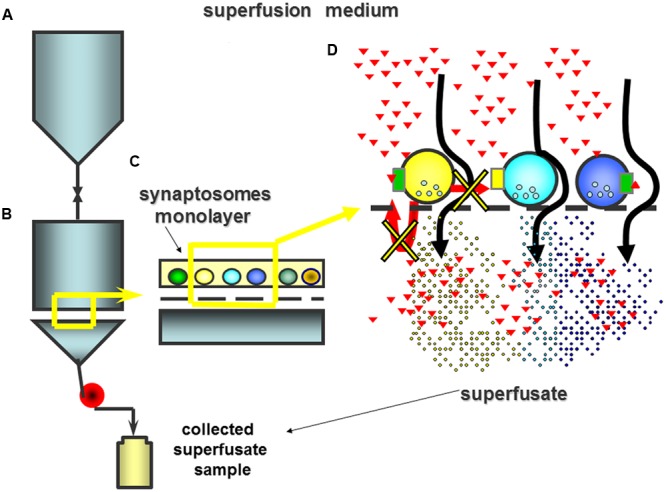
**The superfusion apparatus consists of several (up to 24) units composed of an upper reservoir **(A)** where the medium is thermostated up to 37°C and from which it is rapidly delivered to the lower chamber of superfusion **(B)**.** Synaptosomes are stratified as a monolayer on micro-porous filter **(C)**. In the lower chamber, the superfusion medium is continuously recalled by means of a peristaltic pump. The continuous up-down superfusion assures the rapid removal of any endogenous compounds that is actively released from the stratified particles so that any indirect effects is minimized **(D)**. In these experimental conditions receptors only can be activated by molecules exogenously added to the superfusion medium (red triangles).

These innovations assure the almost-complete absence of biophase and, as a consequence, of indirect effects. Actually, during superfusion, the endogenous compounds are immediately removed, so that their concentration at nerve terminals is minimized and the possibility that they can act on adjacent particles is largely impeded. Inasmuch, the stratification of synaptosomes as a monolayer impedes the possibility that the endogenous molecules can act at synaptosomes located below those particles from which they are released. In these conditions, receptor-mediated changes to the transmitter release can only be elicited by compounds exogenously added at the superfusion medium, which activates the receptors in selected synaptosomal subpopulations (**Figure 2**). In particular, agonist-induced control of transmitter outflows can be evidentiated directly, as drug-mediated changes of exocytosis. Differently, the ability of antagonist(s) to modulate release is mainly highlighted as reversal of the agonist-induced releasing effects.

The synaptosomal suspension originating from a selected central nervous system (CNS) region is composed of particles that are isolated from different subfamilies of nerve endings. The selective labeling with radioactive tracers mimicking endogenous transmitters (i.e., [^3^H]noradrenaline; [^3^H]dopamine; [^3^H]serotonin; [^3^H]GABA) as well as the monitoring of the release of endogenous molecules (glutamate, GABA, glycine, and neuropeptides) permits to isolate a given sub-family of nerve terminals from the entire synaptosomal populations, impeding artifacts that may originate from the heterogeneity of the synaptosomal preparation.

By using this experimental approach, the following functional aspects could be investigated:

(1) The existence and the functional role of release-regulating receptors in nerve terminals as well as their pharmacological profile/subunit composition can be defined. These aspects have been extensively discussed in previous reviews (Raiteri and Raiteri, 2000) and do not need further discussion.
(2) The receptors co-localization and receptor-receptor functional cross-talk (Marchi and Grilli, 2010; Grilli et al., 2012; Marchi et al., 2014, 2015).
(3) The existence of metabotropic receptors adopting a constitutively activated conformation. This conformation can transduce intraterminal signallings despite the absence of agonist at the orthosteric binding site of the receptors (see Presynaptic Release-Regulating mGlu1 Autoreceptors in Mouse Cerebellar Nerve Endings and Presynaptic mGlu1 Heteroreceptors and Dopamine Release from Rat Nerve Endings, Mura et al., 2010; Rossi et al., 2013).
(4) The functional activity of positive and negative allosteric modulators (PAM and NAM) acting at both ionotropic and metabotropic receptors. The classic example of PAM is glycine acting at NMDA receptors controlling the release of noradrenaline and dopamine (Pittaluga et al., 2001), peptides (Gemignani et al., 2000) and glutamate (Luccini et al., 2007b; Musante et al., 2011) terminals. Similarly, cyclothiazide favors the AMPA-evoked release of serotonin, dopamine, and noradrenaline (Pittaluga et al., 1997). Last, but not least, the mGlu5 PAM, the compound [(3-Fluorophenyl)methylene]hydrazone-3-fluorobenzaldehyde (DFB), was shown to amplify the mGlu5-mediated potentiation of noradrenaline exocytosis (Luccini et al., 2007a).
(5) The pharmacological profile of molecules having a complex pharmacological profile can be identified. It is the case of the double-faced mGlu2/3 ligand the compound LY541850, which activates mGlu2 receptor subtypes but inhibits mGlu3 receptors (Di Prisco et al., 2016).

## mGlu1 autoreceptors in central nervous system

### Presynaptic Release-Regulating mGlu1 Autoreceptors in Mouse Cortical Nerve Endings

The first work concerning the existence of group I mGlu autoreceptors in cerebrocortical synaptosomes was by Herrero et al. (1992, 1994), who showed that the glutamate release elicited by 50 μM 4-aminopyridine (4-AP), in the presence of low concentrations of arachidonic acid, was significantly increased by the broad group I mGluR agonist (1*S*,3*R*)-1-Aminocyclopentane-1,3-dicarboxylic acid (1S,3R)-ACPD (**Table 1**).

**Table 1:** Pharmacological properties of the mGlu1 and mGlu5 receptor ligands reported in the text.

Ligand	Pharmacological activity	Reported affinity (units)	Pharmacological properties	Applied concentration (μM)
(*1S,3R*)ACPD	mGlu1/5 receptor full agonist	5.5–6.1 (pIC50)	↑ Transmitter release	100–1000
(RS)CHPG	mGlu5 agonist	3.4 (pIC50)	↑ Transmitter release	10–300
3,5 DHPG	mGlu1/5 receptor full agonist	5.4–5.8 (pIC50)	↑ Transmitter release	0.01–100
MPEP	mGlu5 non-competitive antagonist	7.4–7.7 (pIC50)	↓ 3,5 DHPG-evoked transmitter release	0.1–1
CPCCOEt	mGlu1 non-competitive antagonist	5.3 (pKi)	↓3,5 DHPG-evoked transmitter release	1–5
LY367385	mGlu1 competitive antagonist	5.9 (pKi)	↓ 3,5 DHPG-evoked transmitter release	0.1–10

*The table recapitulates the principal properties of the main mGlu1 and mGlu5 receptor ligands reported in the review. The reported affinity are accordingly to IUPHAR/BPS Guide to PHARMACOLOGY.*

Starting from these observations, the exact identity of the autoreceptors belonging to the group I mGlu receptors (whether mGlu1 receptors or mGlu5 receptors or both) became a matter of discussion. The question, however, has long remained elusive, mainly due to the lack of selective ligands. Similarly, the presynaptic location of these receptors was debated because of contradictory results in the literature (Fotuhi et al., 1993; Shigemoto et al., 1993; Romano et al., 1995; Lujan et al., 1996; Hubert et al., 2001; Muly et al., 2003). Most of these publications, however, agreed on the presence of mGlu5 autoreceptors in nerve terminals isolated from different CNS regions of mammals (Sistiaga et al., 1998; Croucher et al., 2001).

As to the mGlu1 receptor subtype, the hypothesis that these receptors also could have a presynaptic location on glutamate nerve endings was first proposed by Reid et al. (1999). Reid et al. (1999) pharmacologically characterized the group I mGlu autoreceptors in cortical synaptosomes. The most interesting observation was that (RS)-2-Chloro-5-hydroxyphenylglycine (CHPG, **Table 1**), a preferential mGlu5 receptor agonist, failed to potentiate the 4-AP-evoked [^3^H]glutamate release from cerebrocortical synaptosomes, while the selective mGluR1 antagonist (RS)-1-Aminoindan-1,5-dicarboxylic acid (AIDA, **Table 1**) abolished the potentiation elicited by the mGlu1/5 broad spectrum agonist, the compound (S)-3,5-Dihydroxyphenylglycine (3,5-DHPG, **Table 1**). These results led to propose that presynaptic mGlu5 receptors did not account for the functional responses observed, but that the group I mGlu receptors under study belonged to the mGlu1 receptors subtype. These observations were largely confirmatory of previous results obtained in microdialysis studies in the parietal cortex of rats showing a significant increases of glutamate release following local administration of both (1S,3R)-ACPD and 3,5-DHPG (Moroni et al., 1998). AIDA, inactive on its own, antagonized the effect of the group I mGluR agonist. The hypothesis of the presynaptic location of mGlu1 receptors, was, however, contrasted by the almost concomitant findings from Sistiaga et al. (1998), showing that the 3,5-DHPG potentiation of glutamate exocytosis could be observed in cortical nerve terminals isolated from mGlu1 receptors-deficient mice. Based on these findings, the authors excluded that mGlu1 autoreceptors could be present in mouse cortical nerve endings. In line with this conclusion, Croucher’s group demonstrated that the 3,5-DHPG-induced facilitation of [^3^H]D-aspartate ([^3^H]D-ASP) release in slices was largely insensitive to mGlu1 receptors antagonists, but it was potently antagonized by selective mGlu5 receptors blockers (Thomas et al., 2000; Croucher et al., 2001; Fazal et al., 2003).

The question concerning the existence and the role of mGlu1 autoreceptors was reconsidered by Musante et al. (2008b). Using the technique of the up–down superfusion of synaptosomes, these researchers confirmed that 3,5-DHPG, inactive on its own on the spontaneous release of [^3^H]D-ASP, significantly facilitated the exocytosis of the radioactive tracer elicited by a mild (12 mM KCl) depolarizing stimulus from mouse cortical synaptosomes. Quite interestingly, the analysis of the concentration-effect relationship of the agonist on the [^3^H]D-ASP exocytosis unveiled a biphasic pattern, where peaks of potentiation were reached when low (0.3 μM) and high (30–50 μM) concentrations of the agonist were applied. Intermediate concentrations elicited a scarce, not significant, facilitation of glutamate exocytosis. The dual effect seemed predictive of the existence of high- and low-affinity binding sites for the agonist. Notably, the 3,5-DHPG-mediated facilitation of glutamate exocytosis elicited by low (0.3 μM) concentration of the agonist (i.e., the high-affinity binding site) was prevented by 2-Methyl-6-(phenylethynyl)pyridine hydrochloride (MPEP, **Table 1**), while the compound 7-(Hydroxyimino)cyclopropa[b]chromen-1a-carboxylate ethyl ester (CPCCOEt, **Table 1**) was inactive; this is compatible with the involvement of mGlu5 autoreceptors. Differently, the positive effect exerted by higher (i.e., 30 μM) concentration of the agonist (the low affinity binding site) was almost insensitive to MPEP, but it was totally prevented by CPCCOEt, suggesting that mGlu1 autoreceptors play a major role. To conclude, these observations confirmed the presence of high-affinity mGlu5 autoreceptors in mouse cortical nerve endings (as already proposed in literature, Sistiaga et al., 1998; Croucher et al., 2001), but also strongly suggested the presence of low-affinity mGlu1 autoreceptors. Accordingly, the MPEP-sensitive, 3,5-DHPG-mediated facilitation of glutamate exocytosis became undetectable in synaptosomes isolated from the cortex of a transgenic mouse lacking the mGlu5 receptor protein; while the CPCCOEt-sensitive, 3,5-DHPG-mediated facilitation of glutamate exocytosis was undetectable in cortical synaptosomes from mice bearing a spontaneous deletion of the mGlu1 receptor protein (i.e., the *crv4/4* mice, Conti et al., 2006).

Metabotropic glutamate1 and mGlu5 receptors are positively coupled to PLC and elicit diacyl glycerol (DAG) and inositol trisphosphate (IP3) production. Data in the literature had shown that activation of phosphoinositide (PI)-coupled mGlu receptors potentiates the release of glutamate (Herrero et al., 1992; Vazquez et al., 1994), through a DAG/PKC-mediated intraterminal pathway (Coffey et al., 1994; Herrero et al., 1994, 1998). Using a mGluR1 deficient mouse model (Conquet et al., 1994). Sistiaga et al. (1998) indicated that nerve terminals isolated from the cortex exhibit all the responses of the PI-coupled mGluRs observed in wild type mice, including the DAG overproduction and the glutamate exocytosis induced by DHPG. Based on these observations, the authors proposed that mGlu5 receptor subtypes, or an unidentified PI-coupled mGlu receptor, could best account for the modulation of glutamate release in mouse cerebro-cortical nerve terminals. The possibility that mGlu1 receptors could participate in the effects above described, however, was not ruled out. More recently, mouse cortical synaptosomes were analyzed for their endogenous inositol-1,4,5-trisphosphate (IP_3_) content following the exposure to a mild depolarizing stimulus in the absence or in the presence of 3,5-DHPG / MPEP (the antagonist was added in order to impede the mGlu5-mediated metabolic effects). The data demonstrated that the mGlu1/5 receptor agonist facilitated the 12 mM K^+^-evoked production of endogenous IP_3_ and that this facilitation was significantly prevented by CPCCOEt. This is consistent with the participation of the mGlu1-induced signaling in the overproduction of the intraterminal second messenger (Musante et al., 2010). Thus, mGlu1 autoreceptors in mouse cortical nerve endings are positively coupled to an enzymatic pathway leading to IP_3_ overproduction and the consequent release of Ca^2+^ ions from intraterminal stores. Whether the mGlu1/5 receptor agonist could trigger the release of endogenous IP_3_ also in the absence of the depolarizing stimulus was not investigated. 3,5-DHPG, however, failed to modify the spontaneous release of glutamate, suggesting that, if a mGlu1/5-induced mobilization of Ca^2+^ ions had occurred in mouse cortical nerve endings, this event was insufficient to elicit glutamate outflow (see Presynaptic mGlu1 Heteroreceptors and Acetylcholine Release from Human Cortical Nerve Endings).

To substantiate these functional results, subsequent experiments were carried out to investigate whether mGlu1 receptor immunoreactivity exists in mouse cortical synaptosomes. As a first approach, the existence of mGlu1 immunoreactivity in the detergent-soluble fraction isolated from cortical synaptosomes was analyzed. Accordingly to expectation, it was found that synaptosomal lysates are immunopositive for both the mGlu1 and the mGlu5 receptor proteins (Musante et al., 2008b). The synaptosomal fraction used in these experiments, however, contains all the synaptosomal membrane proteins, including those from the organelles and vesicles, as well as those from the postsynaptic membrane fragments that are often sealed to the synaptic boutons during synaptosomes preparations and it did not allow to discriminate between the mGlu receptor proteins located presynaptic or postsynaptically. To definitively address this question, an experimental approach of synaptosomal subfractioning to isolate the pre- and the post-synaptic components of the synaptic active zone was applied. The separation of the two components was validated by analyzing the immunoreactivities of proteins that are considered specific markers of the three synaptosomal fractions (i.e., syntaxin1-A for the presynaptic component and, PSD-95 for the postsynaptic component, Musante et al., 2008b). These experiments confirmed the presynaptic location of the mGlu1 receptor protein, showing that, in addition to mGlu5, mGlu1 receptor immunoreactivity was detectable in the Sintaxin-1A positive presynaptic fraction. mGlu1 immunoreactivity was also observed in the PSD-95-positive, postsynaptic component, which is consistent with the proposed localization of mGlu1 receptors at this side of the active synapses (Ferraguti et al., 2008). Finally, considering that the abovementioned approaches do not allow to discriminate among families of nerve terminals, experiments were carried out to ascertain whether mGlu1 immunoreactivity could be detected in glutamatergic nerve endings. Immunochemical studies demonstrated that both receptor proteins were largely co-expressed with Sintaxin1-A used as a selective presynaptic marker and with the vesicular glutamate transporter type 1 protein (VGLUT-1), used as a selective marker of glutamatergic nerve endings. To conclude, these data were largely confirmatory of the existence of mGlu5 autoreceptors on cortical glutamatergic nerve endings, but also supported the existence of mGlu1 autoreceptors on these terminals.

However, it is not clear whether the two mGlu5 and mGlu1 receptors co-localize on the same terminals, or whether they exist on different nerve endings. Albeit the question cannot be definitively answered, some speculation can be proposed on the basis of the data so far available.

**FIGURE 3:**
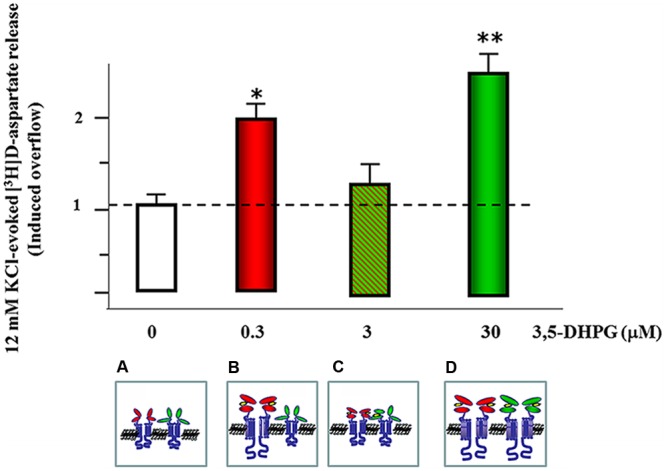
**mGlu1 and mGlu5 receptors co-localize and functionally interact in mouse cortical glutamatergic nerve endings.** mGlu5 receptors (red external domains) represent the high affinity agonist binding sites of the agonist, which is fully activated when synaptosomes are exposed at concentration of 3,5-DHPG as low as 0.3 μM; ^∗^*p* < 0.05 vs. control (empty bar). **(A)** The occupancy of the orthosteric binding sites of these receptors leads to an overt potentiation of glutamate exocytosis. Increasing the external concentration of the agonist, however, also allows the occupancy of the mGlu1 receptors (green external domains) **(B)**. These receptors represent the low affinity binding sites of the agonist and their orthosteric binding sites are fully activated when synaptosomes are exposed at concentration of 3,5-DHPG as high as 30 μM. The exposure of cortical glutamatergic nerve terminals to intermediate concentration of 3,5-DHPG (3 μM) allows the partial occupancy of the orthosteric binding sites of the mGlu1 receptors. We propose that when exposed at concentration of 3,5-DHPG as low as 3 μM, the partial occupancy of the mGlu1 agonist binding sites can negatively reverberate on the mGlu5 receptor component of the mGlu1/mGlu5 receptor/receptor complex, leading to the falls of efficiency of the agonist to potentiate glutamate exocytosis **(C)**. The further increase of the concentration of 3,5-DHPG assures the full occupancy of the orthosteric binding sites in both the receptors, unveiling the maximal facilitation of glutamate exocytosis; ^∗∗^*p* < 0.01 vs. control (empty bar) **(D)**.

The most conservative hypothesis maintains that the mGlu1 and the mGlu5 receptors exist on different subpopulations of cortical glutamatergic nerve terminals. If true, this hypothesis implies that the releasing effects elicited by the two receptors are additive. This, however, was not the case of the mGlu1 and 5 autoreceptors controlling glutamate exocytosis in mouse cortex. Actually, the concentration-response curve of 3,5-DHPG displays a biphasic pattern (**Figure 3**) that seems best interpreted by assuming the colocalization and the functional cooperation of a high-affinity mGlu5 and a low-affinity mGlu1 receptor. This is consistent with recent data suggesting that mGlu receptors can exist either as homomeric or heterodimeric assemblies as well as inter-dimeric complexes (Doumazane et al., 2010). The observation that anti-mGlu1 and anti-mGlu5 immunoprecipitates from mouse cortical synaptosomes were immunopositive for the mGlu5 receptor protein and the mGlu1 receptor protein, respectively (Musante et al., 2010), seems in line with the proposed mGlu1 and mGlu5 physical interaction. Furthermore, the orthosteric mGlu1 receptor antagonist, the compound (S)-(+)-α-Amino-4-carboxy-2-methylbenzeneacetic acid (LY367385, **Table 1**), efficiently prevented the facilitation of glutamate exocytosis elicited by 3,5-DHPG acting at mGlu1 autoreceptors (as indeed expected), but it also proficiently reduced the potentiation of glutamate exocytosis caused by low submicromolar concentration of agonist acting at mGlu5 receptors (Musante et al., 2008b). Again, this observation strengthened the conclusion that the mGlu5 and the mGlu1 receptors are colocalized and possibly are functionally coupled. Actually, since the data available exclude the possibility that LY367385 acts at mGlu5 receptor subtypes, the most conservative hypothesis is that the binding of the mGlu1 receptor orthosteric antagonist at the mGlu1 receptor protein also might activates compensative allosteric mechanism(s) of control of the heterodimeric complex, influencing the efficiency of the binding of the ligand at the other component, i.e., the high affinity mGlu5 receptor protein.

As a matter of fact, differently from the homodimeric assembly, where the impact of the occupancy of the orthosteric binding sites is allosteric to receptor-mediated functions and not simply cumulative, in an heterodimeric complex, as well as in inter-heterodimeric receptor assemblies, the binding of an orthosteric ligand at one component controls either positively or negatively the pharmacological profile of the other binding site, thus leading to unexpected changes in the concentration-effect relationship of the agonist (Delille et al., 2012).

To note, the functional interaction bridging mGlu1 and mGlu5 receptor proteins might also give a rationale for the reduced release efficiency observed when synaptosomes are exposed at concentration of the agonist that are intermediate to those eliciting the maximal releasing effects. For instance, the partial occupancy of the mGlu1 component of the mGlu1/mGlu5 heterodimeric assembly might causes a transient unbalance of the mGlu1/mGlu5 functional cross-talk (**Figure 3**) accounting for the reduced the release efficiency. Further studies are required to better address this hypothesis.

### Presynaptic Release-Regulating mGlu1 Autoreceptors in Human Cortical Nerve Endings

Preclinical studies of animal models have the potential to identify therapeutic targets in humans. It is therefore essential to determine whether corresponding proteins/molecular pathways also exist in humans. The use of fresh human tissue removed during neurosurgery endowed with native receptor proteins and transporters represents a unique approach to evaluate the pharmacological profile of the molecular structures identified in rodents. This approach could lead to the identification of molecular processes underlying the effects of therapeutics in humans, but also consents the validation of the use of the animal models for pharmacological studies.

The existence and the functional role of mGluR1 autoreceptors were investigated in human cortical synaptosomes. The terminals were isolated from human fresh cortical specimens that were removed during neurosurgery to reach deeply located cerebral tumors (Feligioni et al., 2003; Luccini et al., 2007a; Musante et al., 2008a). A study was carried out to verify the existence and the functional role of mGlu1 autoreceptors controlling [^3^H]D-Aspartate release, (Musante et al., 2010). It was demonstrated that 3,5-DHPG in the presence of MPEP (in order to exclude the release component due to the mGlu5 receptors activation) enhanced the K^+^-evoked release of the excitatory amino acid, an effect that was totally abolished by the mGlu1 receptors antagonist CPCCOEt (Musante et al., 2010). Quite interestingly, the 3,5-DHPG releasing activity was paralleled by an overproduction of endogenous IP_3_ in the synaptosomal preparation, which was significantly prevented by the concomitant addition of the mGlu1 receptor antagonist. At the concentration applied, the agonist failed to affect the spontaneous release of preloaded [^3^H]D-Asp; this is consistent with the idea that in human cortical nerve terminals the metabolic effect(s) elicited by the receptor was insufficient to promote “*per se*” transmitter exocytosis. Finally, mGlu1 receptor immunoreactivity was detected in the lysates from human cortical nerve terminals (Musante et al., 2010). Altogether, these observations allowed the following conclusions: (i) human neocortical glutamatergic nerve endings are endowed with release-regulating mGlu1 autoreceptors, the activation of which favors glutamate exocytosis through a cascade of events that promote the release of Ca^2+^ ions from intraterminal IP_3_-sensitive stores; and (ii) mouse cortical glutamatergic nerve endings are an appropriate model to predict the functional activity of mGlu1 receptor bioactive compounds in humans.

### Presynaptic Release-Regulating mGlu1 Autoreceptors in Mouse Hippocampal Nerve Endings

At the hippocampal level, mGlu1 receptors have been implicated in synaptic transmission and plasticity, neuronal excitability, and firing synchronization (Ferraguti et al., 2008). The existence at the presynaptic level of mGlu1 autoreceptors and their role in controlling glutamate exocytosis in this CNS region is an ongoing matter of discussion. The first demonstration of a potential role of mGlu receptors belonging to the first group in the facilitation of glutamate release from hippocampal terminals was provided by Vazquez et al. (1994). These authors demonstrated that the broad-spectrum agonist ACPD greatly potentiated the Ca^2+^-dependent release of endogenous glutamate evoked by a mild depolarization. Quite interestingly, the ACPD-induced facilitation of glutamate exocytosis became detectable only when arachidonic acid was added concomitantly to the depolarizing stimulus. The positive allosteric activity elicited by the fatty acid was proposed to rely on intraterminal facilitatory effect(s) that paralleled the activation of the mGlu receptors complex. Starting from 1994, using experimental approaches other than synaptosomes, some indications of the respective roles of mGlu5 and mGlu1 receptors in controlling glutamate transmission were provided. In the CA1 neurones of mGlu1-deficient mice, the group I mGlu receptor agonist ACPD elicited excitatory effects similar to those observed in control mice (Aiba et al., 1994; Conquet et al., 1994), but not in mGlu5 deficient mice (Lu et al., 1997; reviewed by Bordi and Ugolini, 1999). This latter observation, however, was not confirmed in a subsequent study (Hsia et al., 1995). Inasmuch, in mGlu1 mutant mice, LTP in the CA3 region was largely reduced (Conquet et al., 1994). Concomitantly, Gereau and Conn (1995) proposed a presynaptic role of mGlu receptors with a group I-like pharmacology in the control of both excitatory and inhibitory synaptic transmissions in the CA1 region. Similar results were obtained by Manzoni and Bockaert (1995). The dual role of the group I mGlu receptors on the release of glutamate in rat hippocampal synaptosomes were then investigated by Rodriguez-Moreno et al. (1998, but see also Vazquez et al., 1995). In an attempt to reconciliate both facilitation and inhibition of glutamate exocytosis the authors proposed that the mGlu-induced facilitation of glutamate release elicited by mGlu receptors with a group I-like pharmacology (Gereau and Conn, 1995) is desensitized by increasing the ambient glutamate. This results from a shift of the coupling of these receptors to an alternative inhibitory intraterminal pathway. Few years later, Mannaioni et al. (1999), proposed that the DHPG-induced increase of the neuron excitability in the hippocampus is mainly due to activation of mGlu5 autoreceptors. Soon after, mGlu1 receptors were also suggested to be located presynaptically in selected regions of rat hippocampus, where their activation suppresses, instead of potentiating, excitatory transmission (Mannaioni et al., 2001). Finally, Smolders et al. (2004) proposed the existence of mGlu1a autoreceptors tonically activated by the ambient glutamate level in the hippocampus. Further investigation is required to confirm the respective contribution of mGlu5 and mGlu1 receptor subtypes to the facilitation of glutamate exocytosis in this CNS region.

### Presynaptic Release-Regulating mGlu1 Autoreceptors in Mouse Cerebellar Nerve Endings

Although the literature remains controversial regarding the existence of mGlu1 receptors in different CNS regions, there is a large consensus on the dominant presence of mGlu1 receptors in the cerebellum. In this CNS region, mGlu1 receptors immunoreactivity is mainly found at the excitatory inputs onto Purkinje cells, particularly in the perisynaptic region of their dendritic spines onto which climbing fibers and parallel fibers make synaptic contact. At this level, however, little mGlu1 receptor expression was detected in parallel fiber terminals (Nusser et al., 1994; Mateos et al., 2000), allowing the conclusion that mGlu1 receptors are preferentially located postsynaptically (Ferraguti et al., 2008). The postsynaptic mGlu1 receptors were proposed to play the major role in controlling cerebellar LTD (Ito and Kano, 1982; Linden and Connor, 1993; Ito, 2002).

Rossi et al. (2013), a study was carried out to investigate whether mGlu receptors belonging to the first group control the release of glutamate from cerebellar synaptosomes. Micromolar 3,5-DHPG failed to affect the spontaneous release of glutamate (measured as release of preloaded [^3^H]D-aspartate) from mouse cerebellar isolated nerve endings, but significantly potentiated the Ca^2+^-dependent exocytosis of the radioactive tracer elicited by a mild depolarizing stimulus (12 mM KCl). The 3,5-DHPG-evoked facilitation of glutamate exocytosis was prevented by the mGlu1 receptor antagonist CPCCOEt, while MPEP was devoid of activity. Based on these functional observations, it was proposed that mGlu1 autoreceptors exist in cerebellar synaptosomes, and that their activation potentiates glutamate exocytosis. Accordingly, (i) lysates from cerebellar synaptosomes were found to be endowed with mGlu1 receptor proteins in immunochemical studies, while the mGlu5 signaling was almost absent, and (ii) the CPCCOEt-sensitive 3,5-DHPG-induced facilitation of glutamate exocytosis could not be observed in the *crv4/4* mice (Conti et al., 2006). Taking into account that the synaptosomal population isolated from the cerebellum mainly consists of pinched off particles from parallel fiber boutons, and to a lower extent, from climbing fiber terminals (Cervetto et al., 2010), it is possible that mGlu1 receptors are mainly expressed in parallel fibers processes. The results obtained in immunocytochemical studies aimed at dissecting the respective localization (presynaptic vs. postsynaptic) of the mGlu1 receptor protein at the parallel fibers – Purkinje cells synapses (see Ferraguti et al., 2008 for discussion), however, contrast this conclusion. Further studies are needed to better address this point.

Surprisingly, the mGlu1 allosteric antagonist CPCCOEt significantly reduced on its own the 12 mM KCl-evoked exocytosis of preloaded [^3^H]D-aspartate. The observation was unexpected since in the up–down superfusion model, antagonists are predicted to be devoid of activity and to be unable to control transmitter release (The Up–Down Superfusion of a Thin Layer of Synaptosomes: Some Experimental Considerations). The inhibitory effect exerted by CPCCOEt on the depolarization-evoked glutamate outflow was not observed in synaptosomes isolated from the cerebellum of mouse lacking the mGlu1 receptor proteins (i.e., the *crv4/4* mice, Conti et al., 2006), thus supporting the conclusion that mGlu1 receptors were involved in the CPCCOEt-mediated control of glutamate exocytosis. Inasmuch, the amount of glutamate released from cerebellar terminals isolated from *crv4/4* mice was significantly lower than that observed from control mice, suggesting that a component of the glutamate exocytosis was strictly dependent on the presence of mGlu1 receptors in nerve terminals (Rossi et al., 2013).

It is known that mGlu receptors possess a typical structure that is typified by the presence of the extracellular Venus Fly Trap (VFT) domain. This sequence can exist in both the open and the closed conformations and the shift from one to the other conformation dictates the receptor activity. The closed conformation occurs when the orthosteric agonist binds to the VFT of the receptor subunit, allowing the coupling to the G protein and the metabolic signaling. In recent years, studies have shown that the VFT domain of the mGlu1 receptor can adopt the closed conformation also in the absence of the orthosteric agonist (De Blasi et al., 2001). In this form, the receptor can trigger the G protein-mediated cascade of events.

In an attempt to give a rationale for the inhibitory effect exerted by CPCCOEt on the depolarization-evoked release of glutamate, Rossi et al. (2013) proposed that in cerebellar terminals mGlu1 receptors located in glutamatergic terminals can spontaneously adopt the closed conformation influencing glutamate exocytosis. If this is the case, one might conclude that the constitutively activated conformation of receptors in nerve endings is retained by particles isolated *ex vivo* and became detectable in “*in vitro*” studies. This aspect is particularly relevant and deserves further studies.

### Presynaptic Release-Regulating mGlu1 Autoreceptors in Mouse Spinal Cord Nerve Endings

At the spinal cord level, activation of group I mGlu receptors has been proposed to favor glutamate release, then contributing to nociceptive plasticity and excitotoxic events that follow spinal cord injury (Lefebvre et al., 2000; Mills et al., 2001; Lorrain et al., 2002). The mGlu5 receptor subtype was proposed to play the major role in these events (Hu et al., 2007; Hu and Gereau, 2011), but the participation of mGlu1 receptor subtypes was not excluded (Mills et al., 2001).

In order to address the question of whether mGlu5/1 heteroreceptors exist in spinal cord glutamatergic terminals and their relative contribution to the control of glutamate release, Giribaldi et al. (2013) monitored the spontaneous release of this transmitter from mouse spinal cord glutamate nerve endings that were exposed in superfusion to the group I mGlu receptor agonist 3,5-DHPG. Differently from all the other CNS regions so far described, the authors found that the spontaneous release of glutamate (measured as release of preloaded [^3^H]D-aspartate) from synaptosomes isolated from the entire spinal cord was significantly potentiated by 0.3–30 μM 3,5-DHPG. As already discussed (see Presynaptic Release-Regulating mGlu1 Autoreceptors in Mouse Cortical Nerve Endings), the observation that activation of metabotropic receptors positively coupled to PLC translocation and membrane PI can elicit glutamate release implies that the Ca^2+^ ions mobilization from the intraterminal stores that follows the activation of these receptors is sufficient enough to trigger transmitter exocytosis (see Longordo et al., 2006; Musante et al., 2008a). Accordingly, exposure of spinal cord synaptosomes to the mGlu1/5 agonist elicited an over-production of IP_3_, which was paralleled by a significant mobilization of Ca^2+^ ions in the cytosol. Inasmuch, blockade of the IP_3_-sensitive receptors with Xestospongin-C prevented the releasing activity (Giribaldi et al., 2013). The existence of mGlu1 and mGlu5 receptor proteins in glutamatergic nerve endings was further supported by immunochemical studies showing that synaptosomal lysate were positive for mGlu1 and mGlu5 immunostaining and that mGlu1 and mGlu5 positivity in synaptosomes was co-expressed with VGlut1 immunoreactivity (Giribaldi et al., 2013). The 3,5-DHPG-induced facilitation of glutamate outflow was largely prevented either by the mGlu1 receptor antagonist CPCCOEt or by the mGlu5 receptor antagonist MPEP, although the two inhibitory effects were not additive when the antagonists were added concomitantly.

The finding that either the selective mGlu1 or the mGlu5 receptor antagonists could prevent the 3,5-DHPG-induced facilitation of glutamate release, suggests that the facilitation of glutamate exocytosis lays on the activation of both receptor subtypes, which are possibly functionally coupled in a heterodimeric assembly or in a dimeric association of dimers. Actually, as already discussed (see Presynaptic Release-Regulating mGlu1 Autoreceptors in Mouse Cortical Nerve Endings), the occupancy of one orthosteric binding site by an agonist in a heterodimeric assembly is expected to modify the second orthosteric binding site. Whatever the receptor organization (mGlu1/mGlu5 heterodimeric assembly or association of mGlu1 and mGlu5 homodimers) it might be predicted that glutamate acting at one receptor subtype should interfere with the binding of the agonist at the hortosteric binding site of the complementary receptor protein, as indeed observed, then controlling the receptor-mediated events. This kind of mGlu1/mGlu5 receptor/receptor interaction does not represent a novelty. Actually, previous studies had shown that mGlu1 and mGlu5 receptors could be mutually exclusive in cells (Benquet et al., 2002) and in nerve terminals (Feligioni et al., 2003).

## mGlu1 heteroreceptors in central nervous system

### Presynaptic mGlu1 Heteroreceptors and GABA Release from Mouse Cortical and Hippocampal Nerve Endings

The existence and the functional role of mGlu1 heteroreceptors controlling GABA release in distinct CNS regions (i.e., in the cortex and in the hippocampus) have been largely debated and their involvement still remains undocumented. It had been shown that selective mGlu1 receptor antagonists are neuroprotective, since they enhance GABAergic transmission in the hippocampus (Battaglia et al., 2001; Pellegrini-Giampietro, 2003; Ferraguti et al., 2008). The mechanism underlying the control of GABA release was proposed not to involve the activation of mGlu1 receptors located on GABAergic nerve terminals, but rather was suggested to depend on an indirect cascade of events leading to a reduced activation of inhibitory CB1 receptors controlling GABA exocytosis. It has been further proposed that the blockade of mGlu1 receptors leads to a reduced production of DAG, (i.e., the second messenger that could be enzymatically transformed into the endogenous acyl-glycerol 2), one of the endogenous agonists at cannabinoid receptors. Blockade of mGlu1 receptors, therefore, might lead to a reduced production of the endocannabinoid, diminishing the probability that the inhibitory CB1 receptors located on GABAergic nerve terminals could be activated (Pellegrini-Giampietro, 2003; Ferraguti et al., 2008). If this is the case, the CB1-mediated inhibitory signaling on GABAergic terminals is diminished, favoring therefore GABA release. Well in line with the hypothesis of an indirect mGlu1-mediated control of GABA exocytosis, it was shown that 3,5-DHPG cannot modify neither the spontaneous nor the KCl-evoked release of endogenous GABA or preloaded [^3^H]GABA from nerve terminals isolated from the cortex and the hippocampus of adult mice (Musante et al., 2010; Zucchini et al., 2013). Inasmuch, it was demonstrated that both CPCCOEt and MPEP also were unable to modify the release of GABA from both synaptosomal preparations, ruling out the possibility that mGlu1 and mGlu5 receptors adopting the constitutively activated form are not present on these terminals.

### Presynaptic mGlu1 Heteroreceptors and Glycine Release in Rodent Central Nervous System

Besides GABA, glycine has a main inhibitory role in CNS of mammals, and it participates in the control of nociception and the processing of pain. De Novellis et al. (2002) provided evidence of the role of mGlu1 receptors to control the release of glycine at the periaqueductal level in awake, freely moving rats. In this study, brain microdialysis experiments were performed to investigate the impact of mGlu receptors ligands on the bioavailability of glycine in dialysate samples. The authors provided clearly evidence that activation of mGlu1 receptors account for the increased release of glycine in the periaqueductal gray (PAG) nucleus. The exact location of these receptors remains, however, to be established.

### Presynaptic mGlu1 Heteroreceptors and Acetylcholine Release from Human Cortical Nerve Endings

The existence of mGlu1 heteroreceptors on cholinergic terminals has been scarcely investigated. The data available in the literature originate from studies aimed at clarifying the impact of the HIV-1 viral protein Tat on central neurotransmission in mammal CNS. The viral protein was reported to affect both the spontaneous and the depolarization-evoked release of selected transmitters (i.e., noradrenaline, glutamate, GABA, and acetylcholine, Neri et al., 2007) from nerve terminals isolated from rodents and human CNS (Feligioni et al., 2003; Longordo et al., 2006). In most cases, the control of transmitter outflow exerted by the viral protein was found to involve the mGlu receptors belonging to the group I. In particular, as the release of acetylcholine is concerned, it was demonstrated that activation of both mGlu1 and mGlu5 heteroreceptors underlies the Tat-induced releasing activity in human cortical cholinergic nerve endings. This conclusion relied on the finding that the Tat-releasing activity was efficiently abrogated by CPCCOEt as well as by MPEP. The two antagonists efficiently abrogated on their own the Tat-mediated releasing activity, but the antagonism did not further increase when they were added concomitantly, which is compatible with the idea that the two receptors were reciprocally exclusive in controlling acetylcholine outflow. The results obtained with the antagonists support the idea that the receptors co-localize on the same terminal and their signaling converge and control a common intraterminal pathway (see Presynaptic Release-Regulating mGlu1 Autoreceptors in Mouse Spinal Cord Nerve Endings). In line with the prediction that mGlu1/5 receptor(s) exist in human cortical cholinergic nerve endings, 3,5-DHPG mimicked Tat in promoting acetylcholine release as well as the endogenous IP_3_ production. Both events were prevented when both CPCCOEt and MPEP were added independently (Feligioni et al., 2003). In an attempt to propose an animal model suitable to study the mGlu1/5 heteroreceptors controlling acetylcholine release, the possibility that Tat and 3,5-DHPG could evoke the release of [^3^H]ACh from rodent cortical nerve endings was also investigated (Feligioni et al., 2003). Unexpectedly, Tat, but not 3,5-DHPG, elicited a marked release of the transmitter in this synaptosomal preparation. The Tat-evoked [^3^H]ACh release was insensitive to mGlu1/5 receptor antagonist, and it did not involve the binding of endogenous IP_3_ at the IP_3_ receptors located on the endoplasmic reticulum. Altogether these observations led to the conclusion that receptors other than mGlu1/5 ones, coupled to different *intraterminal* pathways, account for the releasing activity of the viral protein in rodent terminals. To conclude, these observations led to propose that mGlu1 and mGlu5 heteroreceptors exist on human cholinergic cortical nerve terminals. These receptors elicit an IP_3_-mediated mobilization of Ca^2+^ sufficient to trigger the exocytotic-like release of preloaded [^3^H]ACh. Based on the results obtained with selective group I mGlu antagonist, it seems therefore possible to postulate that the two receptor subtypes (mGlu1 and mGlu5 receptors) are colocalized on the same terminals and cooperate to release preloaded [^3^H]ACh. The expression of these receptors appears to be species-specific, since the releasing activity exerted by 3,5-DHPG could not be replicated in rodent cortical nerve endings. Notably, mGlu1 and mGlu5 receptors were also reported to regulate the excitability of cholinergic interneurons in the striatum (Bonsi et al., 2008). Whether the two receptors colocalize or they exist on different neurons was not elucidated, but they were found to differently modulate the firing of these neurons. In particular, mGlu5 receptors were reported to rapidly desensitize after repetitive stimulation, while mGlu1-mediated effects became detectable once mGlu5 receptors were blocked (Bonsi et al., 2008 and references therein). Further studies are required to better describe the system.

### Presynaptic mGlu1 Heteroreceptors and Noradrenaline Release from Human and Rat Nerve Endings

The majority of noradrenergic terminals and varicosities fail to make synaptic contacts, although they possess the prerequisites of transmitter release (i.e., vesicles and mitochondria). They release the transmitter into the extrasynaptic space that reaches postsynaptic targets through the volume diffusion. The release of noradrenaline is controlled by endogenous ligands acting at receptors located presynaptically on these structures. These receptors could be either autoreceptors (usually α2 receptors) or heteroreceptors. Heteroreceptors sense transmitters that reach noradrenergic boutons by volume diffusion. Among these heteroreceptors, studies have shown the existence and the functional role of glutamate receptors (Fink et al., 1989; Pittaluga and Raiteri, 1990; Pittaluga et al., 1997, 2001, 2005; Grilli et al., 2009).

As to the mGlu receptors belonging to the first group, their presence in rat hippocampal noradrenergic nerve terminals was first addressed by Parodi et al. (2006). The authors provided functional observations indicating that presynaptic release-regulating mGluRs of the group I, subtype 5, exist on noradrenergic axon terminals. The activation of these receptors with 3,5-DHPG alone cannot elicit a releasing activity. At a first glance, the lack of efficacy of 3,5-DHPG in unveiling the release of preloaded [^3^H]noradrenaline ([^3^H]NA) from noradrenergic particles might suggest that these terminals do not possess group I mGlu receptors. This conclusion, however, was ruled out by the observation that a 3,5-DHPG-mediated releasing activity became evident when synaptosomes were concomitantly exposed to mild depolarizing stimuli, such as veratrine or nicotine, acting at nicotine heteroreceptors. Interestingly, in both cases, the 3,5-DHPG-mediated facilitation of noradrenaline exocytosis was prevented by MPEP, while CPCCOEt was ineffective, suggesting that mGlu5 receptors are mainly involved.

The finding that 3,5-DHPG could modulate both events deserves some comments. Veratrine is an alkaloid that elicits transmitter exocytosis by activating Na^+^ channels and by allowing the opening of the Voltage Operated Calcium Channels (VOCCs). Nicotine elicits the exocytotic Ca^2+^-dependent release of preloaded [^3^H]NA from rat hippocampal nerve endings by activating α3β4 and possibly α3β2 nicotinic receptors (nAChRs, Marchi and Grilli, 2010). Both nAChRs are highly permeable to Na^+^ ions, whose influx into synaptosomal particles causes the depolarization of the synaptosomal plasma membranes and the consequent activation of VOCCs. The positive effect exerted by 3,5-DHPG on both depolarizing stimuli, therefore, could rely on a common cascade of event(s) that triggers additive increased bioavailability of Ca^2+^ ions in the cytosol; this in turn promotes the Ca^2+^-dependent mechanism of release of the catecholamine. For instance, by favoring the hydrolysis of membrane phosphoinositide, mGlu1/5 receptors can promote the production of endogenous IP_3_, which then favors the release of Ca^2+^ from the IP_3_-sensitive stores in the endoplasmic reticulum. Accordingly, the 3,5-DHPG-mediated facilitation of noradrenaline exocytosis was prevented by Xestospongin-C, a blocker of the IP_3_-sensitive receptors.

Assuming that all the noradrenergic nerve terminals in rat hippocampus are endowed with mGlu5 heteroreceptors, 3,5-DHPG should be expected to reinforce in a MPEP-sensitive manner the releasing activity elicited by different Na^+^-dependent depolarizing stimuli. In line with this expectation, Luccini et al. (2007a) found that 3,5-DHPG significantly potentiates the releasing activity elicited by the activation of NMDA heteroreceptors on rat hippocampal noradrenergic nerve endings. In this case, however, the effect of 3,5-DHPG was prevented by the concomitant application of MPEP and CPCCOEt, while the two antagonists were inactive when added alone. These observations suggests that: (i) mGlu1 heteroreceptors also exist on rat hippocampal noradrenergic nerve terminals, and (ii) mGlu1 and mGlu5 receptors compensate one for the other in reinforcing the NMDA-mediated releasing activity. Again, the observation that the mGluR-induced facilitation of the NMDA-releasing activity is prevented by a combination of both of the two group I mGlu antagonists tends to exclude the possibility that the two receptors exist on different noradrenergic nerve terminals. Differently, it strongly supports the co-localization of mGlu1 and mGlu5 heteroreceptors on noradrenergic nerve endings bearing NMDA heteroreceptors. To further complicate the scenario, data in the literature had shown that nicotine receptors and NMDA receptors colocalize on rat hippocampal noradrenergic nerve endings, with the nicotine receptors being permissive to NMDA–mediated functions in ionic conditions that are expected to impede NMDA-mediated releasing activity (i.e., in the presence of Mg^2+^ ions, Marchi and Grilli, 2010).

Thus, it appears that in noradrenergic terminals an intricate combination of receptor-receptor functional interactions could take place, allowing the functional recruitment of different mGlu receptor subtypes, depending on the depolarizing stimuli applied. The most conservative hypothesis is that mGlu1 and mGlu5 receptors localize on distinct regions of the plasma membranes of rat hippocampal nerve endings, where they can be differently recruited by local depolarization to interact with co-localized ionotropic receptors. According to this view, receptors present in noradrenergic synaptosomal plasma membranes do not necessary cross-talk, at least by a functional point of view, with group I mGluRs. It is the case, of the cyclothiazide-sensitive GluA2-containing AMPA heteroreceptors, which exist on rat hippocampal noradrenergic nerve endings. Previous studies had shown that these receptors traffic in-out synaptosomal plasma membranes in a constitutive manner (Pittaluga et al., 1997, 2005, 2006). Although these receptors are associated to ionic channels permeable to monovalent cation, their activation did not unveil the mGlu1/5 receptor-mediated releasing activity, suggesting that the receptors are not functionally coupled (Longordo et al., 2006). The low percentage of noradrenergic nerve endings/varicosities in the rat hippocampal synaptosomal fraction does not allow biochemical studies aimed at isolating and identifying the receptor protein localized on this synaptosomal subpopulation. The hypothesis so far described therefore could not be confirmed with immunohistochemical investigations. However, the functional cross-talk bridging NMDA receptors and mGlu1/5 receptors was also demonstrated to take place in human neocortical noradrenergic nerve terminals, supporting the use of the rodent model as a suitable approach to study the amine transmission in human (Luccini et al., 2007a).

### Presynaptic mGlu1 Heteroreceptors and Dopamine Release from Rat Nerve Endings

The existence and the role of group I mGlu receptors in dopaminergic neurones has been largely investigated and the distribution and role of these receptors has been described by Conn et al. (2005). mGlu1 receptors were propose to exist on dopaminergic fibers which converge with corticostriatal glutamatergic terminals on medium spiny neurones. Whether these receptors are present in dopaminergic striatal nerve endings, and if their activation directly modifies dopamine release, was scarcely investigated. So far, mGlu5 and mGlu1 heteroreceptors controlling the release of preloaded [^3^H]dopamine ([^3^H]DA) were shown to exist on caudate putamen synaptosomes preloaded with the tritiated radioactive tracer. In particular, MPEP and CPCCOEt significantly inhibited the release of [^3^H]DA elicited by a mild depolarizing stimulus. This observation supports the idea that in these terminals both receptors could have adopted a constitutive active conformation (Mura et al., 2010). Further studies are, however, required to address this issue.

## Conclusion

**FIGURE 4:**
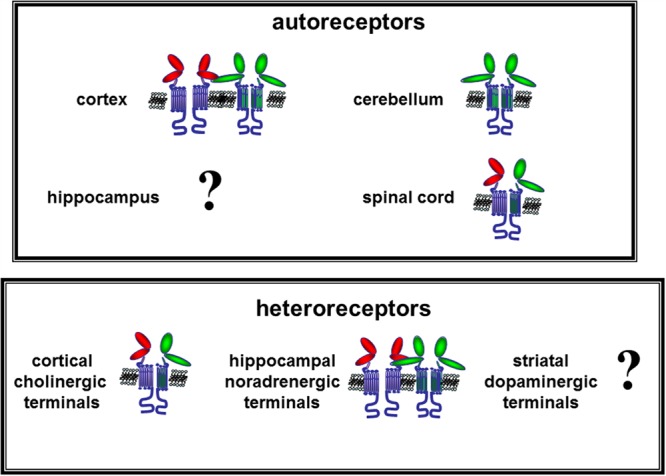
**Hypothesis on the mGlu1 auto and heteroreceptors assembly in nerve terminals from different CNS regions and their functional interaction with mGlu5 receptors.** The data available in literature suggest that low affinity mGlu1 (green external domains) receptors exist in human and mouse cortical nerve endings where they are physically linked and functionally coupled to co-localized high affinity mGlu5 (red external domains) autoreceptors. Low affinity mGlu1 autoreceptors also exist in cerebellar glutamatergic synaptosomes, which adopt a constitutively activated conformation that is retained by isolated cerebellar synaptosomes. mGlu1 autoreceptors are present in spinal cord glutamatergic nerve endings where they are coupled to mGlu5 autoreceptors in a functional association that is mutually exclusive. Finally, the existence of mGlu1 autoreceptors in hippocampal glutamatergic nerve endings has been postulated but direct evidence supporting this notion are not so far available. Further studies are required to better characterize these receptors. As the mGlu1 heteroreceptors are concerned, evidence demonstrating the existence of mGlu1 receptors on GABAergic terminals is so far lacking. Differently, mGlu1 heteroreceptors exist on human cholinergic nerve endings. These receptors are coupled to mGlu5 heteroreceptors; the two receptors substitute one for each other and their activation elicits acetylcholine release. mGlu1/mGlu5 heteroreceptors also exist on human and mouse noradrenergic nerve endings. These receptors are unable on their own to release the amine but positively control the releasing activity of co-localized NMDA receptors.

The present work aimed to review the evidence recently accumulated in favor of the existence and the role of presynaptic mGlu1 receptors as modulators for the release of transmitters from nerve terminals in mammalian CNS. These data suggest a complex scenario in which mGlu1 receptors exist on different synaptosomal subpopulations in different CNS regions. The activation of presynaptic mGlu1 receptors exerts distinct actions depending on the nerve terminal subtype involved, but mainly rely on the activation of intraterminal pathways positively coupled to the hydrolysis of membrane phosphoinositide. The concomitant application of a mild depolarizing stimulus is in most cases essential to acquire the mGlu1-mediated releasing activity. mGlu1 autoreceptors can adopt a constitutively activated conformation, which functional role deserves further investigation. mGlu1 receptors colocalize and are functionally coupled to mGlu5 receptors, by means of complex interactions that, depending on the nerve terminals subfamily, make the two receptors mutually exclusive, mutually compensatory, or independent one from each other. mGlu1/mGlu5 receptor/receptor interaction is strictly region- and transmitter- dependent, as summarized in **Figure 4**. The receptor models here described represent suitable experimental paradigm(s) for the study of new agents acting at both the orthosteric and the allosteric binding sites of mGlu1 receptors, allowing the advance in the knowledge of their role in patho-physiological conditions.

## Author Contributions

The author confirms being the sole contributor of this work and approved it for publication.

## Conflict of Interest Statement

The author declares that the research was conducted in the absence of any commercial or financial relationships that could be construed as a potential conflict of interest.
